# A Cross-Scale Neutral Theory Approach to the Influence of Obesity on Community Assembly of Human Gut Microbiome

**DOI:** 10.3389/fmicb.2018.02320

**Published:** 2018-10-29

**Authors:** Wendy Li, Yali Yuan, Yao Xia, Yang Sun, Yinglei Miao, Sam Ma

**Affiliations:** ^1^Computational Biology and Medical Ecology Lab, State Key Laboratory of Genetic Resources and Evolution, Kunming Institute of Zoology, Chinese Academy of Sciences, Kunming, China; ^2^College of Clinical Medicine, Lanzhou University, Lanzhou, China; ^3^Kunming College of Life Science, University of Chinese Academy of Sciences, Kunming, China; ^4^Department of Gastroenterology, The First Affiliated Hospital of Kunming Medical University, Yunnan Institute of Digestive Disease, Kunming, China; ^5^Center for Excellence in Animal Evolution and Genetics, Chinese Academy of Sciences, Kunming, China

**Keywords:** obesity, Hubbell neutral theory of biodiversity, Sloan's neutral model for microbes, niche theory, community assembly, species abundance distribution (SAD)

## Abstract

**Background:** The implications of gut microbiome to obesity have been extensively investigated in recent years although the exact mechanism is still unclear. The question whether or not obesity influences gut microbiome assembly has not been addressed. The question is significant because it is fundamental for investigating the diversity maintenance and stability of gut microbiome, and the latter should hold a key for understanding the etiological implications of gut microbiome to obesity.

**Methods:** In this study, we adopt a dual neutral theory modeling strategy to address this question from both species and community perspectives, with both discrete and continuous neutral theory models. The first neutral theory model we apply is Hubbell's neutral theory of biodiversity that has been extensively tested in macro-ecology of plants and animals, and the second we apply is Sloan's neutral theory model that was developed particularly for microbial communities based on metagenomic sequencing data. Both the neutral models are complementary to each other and integrated together offering a comprehensive approach to more accurately revealing the possible influence of obesity on gut microbiome assembly. This is not only because the focus of both neutral theory models is different (community vs. species), but also because they adopted two different modeling strategies (discrete vs. continuous).

**Results:** We test both the neutral theory models with datasets from Turnbaugh et al. (2009). Our tests showed that the species abundance distributions of more than ½ species (59–69%) in gut microbiome satisfied the prediction of Sloan's neutral theory, although at the community level, the number of communities satisfied the Hubbell's neutral theory was negligible (2 out of 278).

**Conclusion:** The apparently contradictory findings above suggest that both stochastic neutral effects and deterministic environmental (host) factors play important roles in shaping the assembly and diversity of gut microbiome. Furthermore, obesity may just be one of the host factors, but its influence may not be strong enough to tip the balance between stochastic and deterministic forces that shape the community assembly. Finally, the apparent contradiction from both the neutral theories should not be surprising given that there are still near 30–40% species that do not obey the neutral law.

## Introduction

Obesity is a complex physiological disorder that is often associated with multi-organ (e.g., cardiac, adipose, muscle, hypothalamic, pancreatic, and hepatic tissue), chronic metabolic, and inflammatory alterations. Obesity may induce some chronic metabolic diseases directly or indirectly, such as type 2 diabetes, atherosclerosis, nonalcoholic fatty liver disease, and gout (Sun et al., 2012; Henao-Mejia et al., 2014). Obesity has become a serious health threat to a growing number of people around the world in the past decades. Obesity epidemic relates to many factors, including not only diet habits, physical activity, and genetic makeup (Ravussin and Ryan, 2018), but also behavioral factors, environmental exposures, social-psychological factors, and reproductive factors (Davis et al., 2018). In addition, its close links with the human gut microbiome have been revealed by more recent studies in the last decade (e.g., Turnbaugh et al., 2006, 2009; Zhao, 2013; Davis et al., 2018). Because of the significant overlap between obesity and the metabolic syndrome, dysbiosis of gut microbiome or shift of the balance, is a phenomenon deserving serious considerations when assessing the elements driving adiposity (Stephens et al., 2018). Several studies showed a significant difference in the ratio of *Firmicutes* to *Bacteroidetes*, where higher *Firmicutes* and lower *Bacteroidetes* were found in obese subjects (Ley et al., 2005, 2006; Turnbaugh et al., 2006, 2009; Armougom et al., 2009; Hildebrandt et al., 2009; Fleissner et al., 2010; Murphy et al., 2010), but exceptions regarding the ratio change were also reported (Schwiertz et al., 2010; Zhang et al., 2010; Zhao, 2013). More recent studies found that the abundance of *Bacteroides thetaiotaomicron* remarkably decreased in obese individuals (Liu et al., 2017), and the ratio of two enterotypes in human gut microbiome (*Prevotella* spp. to *Bacteroides* spp.) has been shown to play a role in predicting the weight loss of people with different diets (Hjorth et al., 2017). Goodrich et al. found that the family *Christensenellaceae* was enriched in individuals with low body mass index (BMI), and the weight is reduced in the recipient mice inoculated with *Christensenella minuta* (Goodrich et al., 2014, 2016a,b). Menni et al. (2017) further assessed the association of gut microbiome composition and change in body weight over time by analyzing the data of 1632 females from “TwinsUK” database including longitudinal BMI data and fecal microbiome data. They demonstrated that *Ruminococcaceae* and *Lachnospiraceae* were associated with lower long-term weight gain, and *Bacterioides* was associated with increased risk of weight gain. In addition, many studies have suggested the lowered gut microbial diversity in obese individuals (Ley et al., 2006; Turnbaugh et al., 2009; Le Chatelier et al., 2013). In spite of the extensive studies on the relationship between gut microbiome diversity and obesity, and several computational models that can help for predicting potential obesity-related microbe (Chen et al., 2017; Huang et al., 2017a,b; Wang et al., 2017), the underlying mechanism has not been addressed to the best of our knowledge.

The mechanisms of species coexistence and biodiversity maintenance in ecological communities have long been a core research theme of community ecology, in which the deterministic niche theory and stochastic neutral theory are well recognized as two most influential. Traditional niche theory maintains that species coexisting in a community must have different niches, and species with the same niche requirements could not stably coexist in long term (Matthews et al., 2014). Although niche theory was supported by many field and laboratory studies, it encountered difficulties in explaining the mechanisms of species coexistence in tropical forests. Hubbell (1997, 2001); Wills et al. (1997) introduced the neutral theory of biodiversity that provided alternative perspectives of species coexistence. Hubbell's neutral theory of biodiversity is an individual-based stochastic dynamic theory that assumes equivalences among interacting species and can be formulated as a dispersal-limited, distribution-sampling model (Etienne, 2005; Alonso et al., 2006; Rosindell et al., 2011, 2012). The latter allows rigorous statistical testing of the neutral theory with the species abundance data (SAD) that can be obtained from field survey (in macro-ecology of plants and animals) or metagenomic sequencing data (in microbial ecology).

In consideration of the unique characteristics of metagenomic sequencing data of microbial species abundance distribution, Sloan et al. (2006, 2007) proposed an alternative neutral model that emphasizes the species-level neutrality in microbial communities. Unlike traditional neutral theories that were calibrated by using “almost complete description of the taxa-abundance distribution for community,” Sloan's model can calibrate itself just with the small-sample microbial data that were collected using molecular approaches since Sloan's model allowed for the difference of competitiveness among species in microbial communities (Sloan et al., 2006, 2007). Another important characteristic of Sloan's model is that it was derived from a continuous diffusion process rather than from a discrete distribution model as that of Hubbell (Sloan et al., 2006, 2007). These two features make Sloan's neutral model a nice complement to Hubbell's neutral model (Hubbell, 2001; Etienne, 2005; Rosindell et al., 2011, 2012).

The neutral theory offers a powerful quantitative tool to identify the forces that shape the gut microbial communities, and the revealed information is crucial for understanding the mechanisms that maintain microbiome diversity and possible influences of diseases/disorders such as obesity on the mechanistic shifts of community assembly. In spite of extensive studies on the relationship between the gut microbiome and obesity, as reviewed previously, whether or not obesity plays a tipping role in “re-assembling” gut microbiota, or exerting a significant influence on the mechanisms of community assembly and diversity maintenance, is still an open question. For example, the test of neutral theory can help to answer the following question: which forces, deterministic host factors such as obesity, or stochasticities in birth, death and migration of gut microbes, are in control of the composition and diversity of gut microbiome. If the former is the case, it suggests that the community is formed through the partition of different niches, occupied by species with different niche requirements, and the exhibited diversity (heterogeneity) at the community level is determined by the deterministic environmental factors that delineate different niches. If the latter is the case, it suggests that the community is essentially a random mix of largely ecological equivalent species, and the exhibited diversity (heterogeneity) is caused by the stochasticities in birth, death and migration of different species. The primary objective of this article is to apply the neutral theories of Hubbell (2001) and Sloan et al. (2006, 2007) for exploring the above question with the dataset from a large-scale, comprehensive study of the human gut microbiome involving 283 overweight, obese and lean individuals, originally reported by Turnbaugh et al. (2009).

## Material and methods

### Dataset description

The 16S r-RNA datasets of gut microbiomes we used to test the neutral theories were first reported in Turnbaugh et al. (2009), and a brief description is presented as follows. A series of fecal samples were collected from 154 individuals, including 31 monozygotic twin pairs, 23 dizygotic twin pairs and their mothers (*n* = 46), and each participant was sampled twice with an average interval between sampling of 57 ± 4 days. A total of 283 fecal samples were taken, including 196 were collected from participants in obesity (BMI > 30 kg/m^−2^), 61 were collected from participants in leanness, and 24 were collected from overweight participants (BMI ≥ 25 and < 30). The datasets of 16S rRNA reads and corresponding species or OTU (operational taxonomic unit) table was obtained by using the 454 FLX platform and subsequent bioinformatics analysis. Each sample corresponds to one row in the OTU table, and was treated as one microbial community. More detailed information on the dataset is referred to Turnbaugh et al. (2009).

### Hubbell's (2001) neutral theory model

Hubbell's neutral theory is an individual-based sampling theory, and offers a biological occurrence mechanism to explain observed species abundance distributions (SADs) in ecological communities. It assumes that all individuals in a saturated local community are ecologically equivalent, which means they have the same rate of birth, death and migration, excluding their random fluctuations. Etienne (2005) developed a sampling formula (distribution) that can be utilized to statistically test the Hubbell' neutral theory with field observation data of SAD, in our case the OTU tables described in the previous section.

Etienne sampling formula (Etienne, 2005) is with the following form:

(1) $$\begin{array}{r} P\left(D|\theta ,m,J\right)=\frac{J!}{\prod_{i=1}^{S}n_{i}\prod_{j=1}^{J}\phi_{j}!}\frac{\theta^{S}}{{\left(I\right)}_{J}}\overset{J}{\underset{A=S}{\sum }}K\left(D,A\right)\frac{I^{A}}{{\left(\theta \right)}_{A}}, \end{array}$$

where *m* is the migration probability, *J* is the total number of individuals in the community, *I* is the number of immigrants that compete with the local community individuals, *S* is the total number of species, θ is the fundamental biodiversity parameter of the formula, *n*_*i*_ is the abundance of species *i*, ϕ_*j*_ is the number of species with abundance *j, D* is the species-abundance distribution containing the abundance of each species, *D* = (*n*_1_*, n*_2_*, …, n*_*s*_).

The immigration rate (probability) *m* is further defined as:

(2) $$\begin{array}{r} m=\frac{I}{I+j-1}, \end{array}$$

*K*(*D, A*) is further defined as:

(3) $$\begin{array}{r} K\left(D,A\right)=\underset{\left\{a_{1},a_{2}\ldots .a_{S}|\overset{S}{\underset{i=1}{\sum }}a_{i}=A\right\}}{\sum }\overset{S}{\underset{i=1}{\prod }}\frac{{\bar{s}}_{\left(n_{i},a_{i}\right)}{\bar{s}}_{\left(a_{1},1\right)}}{{\bar{s}}_{\left(n_{i},1\right)}} \end{array}$$

where *a*_*i*_ is the number of ancestors of the species *i*, and the summation is over *a*_i_ = 1, …, *n*_*i*_ with the restriction that the *a*_*i*_ sum to *A*.$\bar{s}\left(n_{i},a_{i}\right)$ is defined as:

(4) $$\begin{array}{r} \bar{s}\left(n_{i},a_{i}\right)=\underset{\left\{D_{+,i}|a_{i}\right\}}{\sum }\left(\frac{n_{i}!}{\prod_{j=1}^{n_{i}}J^{\phi_{i,j}}\phi_{i,j}!}\right) \end{array}$$

and $\bar{s}\left(n_{i},1\right)$ and $\bar{s}\left(a_{i},1\right)$ are factorials of (*n*_*i*_ −1) and (*a*_*i*_ − 1), respectively (Tavaré and Ewens, 1997).

Then we used the following equation to compare the observed community and neutral theory predicted community:

(5) $$\begin{array}{r} D=-2\ln\;\left(\frac{L_{0}}{L_{1}}\right)=-2\left[\ln\;\left(L_{0}\right)-\ln\;\left(L_{1}\right)\right] \end{array}$$

where *L*_0_ represents the log-likelihood of the null model and *L*_1_ represents the log-likelihood of the alternative model, and *D* is the deviation. The *p-value* was computed *via* an *X*^2^-distribution with the degree of freedom being one.

Etienne (2005) sampling formula is used to test the neutrality of fecal microbial communities through Etienne's *Exact* test of neutrality. The Etienne's “Exact neutrality test,” which is based on the sequential construction schemes, does not require alternative model in hypothesis testing. Therefore, it avoids the discussion of validity of the alternative model in empirical evaluations (Etienne, 2007). In brief, firstly, we apply the maximum likelihood estimation (MLE) method to estimate the parameters of the neutral model. This process was performed using the R package UNTB (available at: https://cran.r-project.org/web/packages/untb/index.html). Secondly, for each sample, we simulated 100 artificial communities (datasets) using the estimated parameters (θ, *I, J*) and then calculated the likelihood for each artificial dataset via Etienne formula, namely *P*_s_. Finally, we compared the mean of the likelihoods (*P*_s_) of 100 artificial datasets for each sample and the likelihood (*P*_0_) of the corresponding observed sample using a Chi-squared test. The null hypothesis is that there is no significant difference between the probability from the observed community and the values computed from the artificial data sets. If no significant difference between *P*_s_ and *P*_0_ were detected, the community would be judged as neutral. The *p*-value of 0.05 (*p* > 0.05) is adopted as the threshold for passing the neutrality test.

### Sloan's (2006) neutral theory model

Sloan et al. (2006) derived an alternative neutral model based on Hubbell's (2001) neutral theory. Sloan's model was aimed to address the difficulty in inferring the taxa-abundance distribution of a microbial community from small metagenomic samples. Sloan's model assumes that the local (or destination) community is saturated with a total of *N*_*T*_ individuals. In the local community, an individual either dies locally or immigrate from the remote (source) community, which occurs at a species-independent rate δ. An immigrant from a source community, with probability *m*, would immediately replace the dead individual, or a local-born member with probability 1–*m* would replace it. Hence, the destination community is assembled/reassembled (formed and developed) through a continuous cycle of immigration, reproduction and death. Further assuming that deaths are uniformly distributed in time, then one death is expected during a period of time *1/*δ. In the meantime, the *i-*th species, whose initial absolute abundance was *N*_*i*_, would either increase by one, stay the same or decrease by one with the probability specified by the following three expressions, respectively.

(6) $$\begin{array}{r} \mathrm{Pr}\left(N_{i}+1/N_{i}\right)=\left(\frac{N_{T}-N_{i}}{N_{T}}\right)\left[mp_{i}+\left(1-m\right)\left(\frac{N_{i}}{N_{T}-1}\right)\right] \end{array}$$

(7) $$\begin{array}{rcl} \mathrm{Pr}\left(N_{i}/N_{i}\right) & = & \frac{N_{i}}{N_{T}}\left[mp_{i}+\left(1-m\right)\left(\frac{N_{i}-1}{N_{T}-1}\right)\right]+\left(\frac{N_{T}-N_{i}}{N_{T}}\right)\\ \left[m\left(1-p_{i}\right)+\left(1-m\right)\left(\frac{N_{T}-N_{i}-1}{N_{T}}\right)\right] \end{array}$$

(8) $$\begin{array}{r} \mathrm{Pr}\left(N_{i}-1/N_{i}\right)=\frac{N_{i}}{N_{T}}\left[m\left(1-p_{i}\right)+\left(1-m\right)\left(\frac{N_{T}-N_{i}}{N_{T}-1}\right)\right] \end{array}$$

Let *x*_*i*_ be the *occurrence frequency* of the *i*-th species in the destination community, i.e., *x*_*i*_ = *n*/*N*, where *n* is the number of local community samples where species *i* occurred and N is the total number of local community samples (Burns et al., 2016), *p*_*i*_ is the occurrence frequency of *i*-th species in the source community, i.e., the counterpart of *x*_*i*_ in the destination community Sloan et al. (2006) showed that *x*_*i*_ should follow the following beta distribution:

(9) $$\begin{array}{r} x_{i}\sim Beta\left[N_{T}mp_{i},N_{T}m\left(1-p_{i}\right)\right]. \end{array}$$

Specifically,

(10) $$\begin{array}{r} \phi_{i}\left(x_{i};N_{T},p_{i},m\right)=cx_{i}^{N_{T}mp_{i}-1}{\left(1-x_{i}\right)}^{N_{T}m\left(1-p_{i}\right)-1}, \end{array}$$

(11) $$\begin{array}{r} c=\frac{\Gamma \left(N_{T}m\right)}{\Gamma \left[N_{T}m\left(1-p_{i}\right)\right]\Gamma \left(N_{T}mp_{i}\right)}, \end{array}$$

where *N*_*i*_ and *N*_*T*_ are the total number of individuals of species *i* and the total number of individuals (of all species) in the local community samples, respectively, *m* is the migration frequency, and ϕ_*i*_ represents the probability density function, rather than the number of species mentioned in Equation 1.

According to Burns et al. (2016), the process for testing Sloan et al. (2006) neutral model can be summarized as the following three steps.

(*i*) Compute *p*_i_ and *x*_*i*_, with both *p*_i_ and *x*_i_, one can fit the beta distribution (Equations. 7, 8) and obtain the estimation of *m*.

(*ii*) Compute the predicted (theoretical) φ_*i*_ the theoretical occurrence frequency of species *i* across all destination community samples, based on *m* and the beta distribution (Equation 8).

(*iii*) Judge whether or not the observed *x*_*i*_ of species *i* falls within its theoretical interval φ_*i*_ predicted from the neutral model, and obtain a list of neutral species whose observed *x*_*i*_ satisfy the prediction from the neutral model.

Unlike Hubbell (2001) neutral theory model, there is not a community level statistic (*p*-value) for testing neutrality with Sloan's model (Sloan et al., 2006, 2007), other than the percentage of neutral or non-neutral species. Obviously, it is not easy to define what “majority” level of the neutral species to designate the whole community as neutral as in the case of Hubbell's model. Another important metric that can be utilized to judge the goodness-of-fitting for Sloan's model is the *R*^2^ or *R*-squared, the coefficient of determination. Another important metric that can be utilized to judge the goodness-of-fitting for Sloan's model is the *R*^2^ or *R*-squared, the coefficient of determination. We use a subjective threshold of *R*-squared = 0.5 as passing the Sloan model test.

## Results and discussion

### Testing the influence of obesity on neutrality at the community level

We tested the neutrality of gut microbial community samples using Etienne sampling formula. The model parameters were estimated using the MLE (maximum likelihood estimation) & LLR (log-likelihood ratio) test, as detailed in Etienne (2005, 2007) and Li and Ma (2016). To perform the LLR test, we compared the log-likelihood of each observed gut microbial community with the average log-likelihood of corresponding simulated communities based on the neutral model, and the *p*-value of the LLR test was listed in the online Supplementary Table S1.

**Figure 1:**
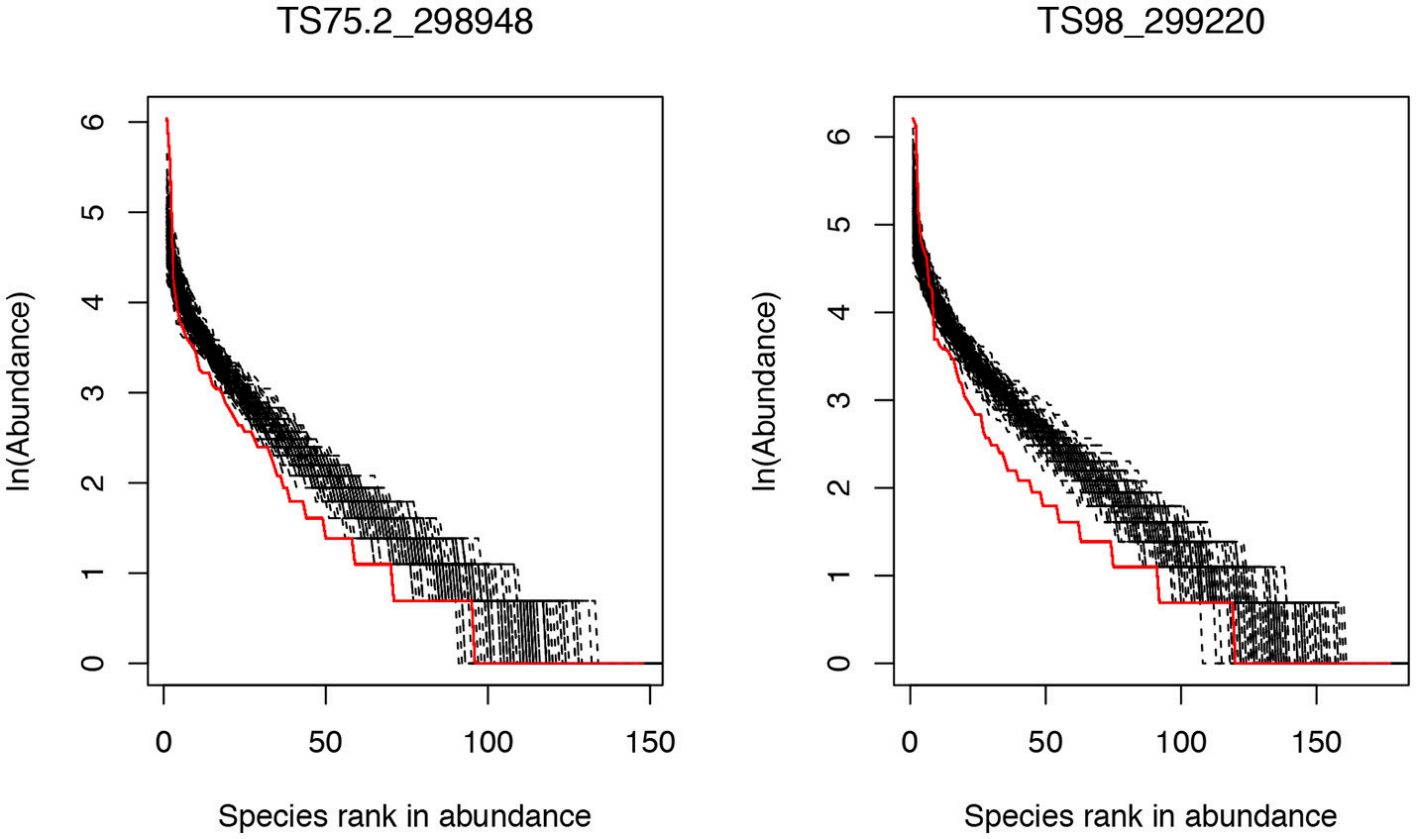
The rank abundance curves of two community samples that successfully passed the neutrality test: the solid red line represents for the observed community and the black dash lines for the simulated communities based on the neutral theory model. The *X*-axis is the species rank order in abundance and *Y*-axis is the abundance of each species in natural logarithm.

The results in Supplementary Table S1 show that there were only 2 gut microbial communities (subject ID: TS75.2_298948 and TS98_299220) out of 283 communities that passed Etienne neutrality test of Hubbell's neutral theory. Both the communities satisfying the neutral community model were sampled from the obese patients, and their neutral model parameters are summarized in the following Table 1. Figure 1 displays the graphs of fitting the neutral theory model to these two communities that passed the neutrality exact test.

**Table 1:** The gut microbial communities passing the neutrality exact test with Etienne sampling formula based on (Hubbell, 2001) neutral theory model^*^.

***Treatment***	**ID**	***J***	***S***	**θ**	***m***	**Log(*L_0_*)**	**Log(*L_1_*)**	***q-*value**	***p-*value**
Obese	TS75.2_298948	1676	148	38.947	0.99997	−86.334	−85.809	1.051	0.3052
	TS98_299220	2602	177	42.752	0.99991	−110.238	−108.549	3.379	0.0660

* *The total number of reads (total individuals) in the sample community (J), the number of species (S), the fundamental biodiversity (θ), the immigration probability (m), log-likelihood of the observed sample [log(L_0_)], log-likelihood predicted by the neutral model (log(L_1_)), and the log-likelihood ratios (q-value and p-value). P-value >0.05 indicates the community satisfies the prediction of Hubbell's neutral theory*.

The test results presented in Supplementary Table S1 and Table 1, as well as Figure 1, revealed that, at the whole community level, the number of communities (only 2 out of 283) passing the neutrality test of Hubbell's neutral theory is negligible. Therefore, the assembly processes of gut microbiota should be dominantly shaped by host environmental effects rather than by stochastic neutral effects such as birth/death stochasticities. While the compositions and diversities of gut microbial communities may be different between obese and healthy people as demonstrated in existing studies (Ley et al., 2006; Liu et al., 2017; Menni et al., 2017), obesity is not strong enough to change the intrinsic mechanisms of the community assembly and diversity maintenance in the gut microbiome. In other words, the structure of gut microbiome is primarily shaped by rather strong deterministic host environment, and stochasticities in gut microbial communities do not play a significant role in shaping the assembly of gut microbiome. Furthermore, obesity as a relatively common health disorder nevertheless, does not change the landscape of gut microbiome assembly.

### Testing the influence of obesity on neutrality at the species level

While the previous section was focused on testing the influence of obesity on gut microbiota neutrality at the whole community level based on Hubbell's (2001) neutral model, here our focus is the neutrality at species level based on Sloan et al. (2006, 2007) neutral model. Because the results from testing Sloan's neutral model may be influenced by samples sizes, we randomly sampled 50 microbiota samples from the lean and obese treatments, respectively, to achieve balanced sample sizes between both the treatments. We further repeated this sampling process 30 times. The averages of the 30 times were taken as the final results of testing Sloan's neutral model (Table 2) and the standard deviations were displayed in Supplementary Table S2.

**Table 2:** The gut microbial species passing the test of Sloan's neutral theory in the gut microbiome of lean and obese treatments^*^.

**Source**	**Destination**	***N***	***m***	***R*^2^**	**Total**	**Neutral (%)**	**Non-neutral (%)**
Lean	Lean	3629	0.043	0.416	1640	65.5	34.5
Obese	Obese	2543	0.063	0.472	1476	68.5	31.5
Lean	Obese	2586	0.032	0.296	1220	58.6	41.4

* *N is the average individuals in destination community, m is the immigration probability, R^2^ is the goodness-of-fitting, total is the total number of species in the treatment, neutral is the percentage of the species within the 95% confidence interval predicted by the neutral model, and non-neutral is the percentage of the species deviating from the neutral model*.

The parameters listed in Table 2 included the average individuals in destination community (*N*), the immigration rate (*m*), the goodness-of-fitting (*R*^2^), and the total number of species in each treatment (*Total*). The column “*Neutral*” in Table 2 listed the percentage of the species within the 95% confidence intervals predicted by the best-fitted neutral model. These species follow Sloan's neutral theory. The column “*Non-neutral”* listed the percentage of the species deviating from the prediction of Sloan's neutral model.

**Figure 2:**
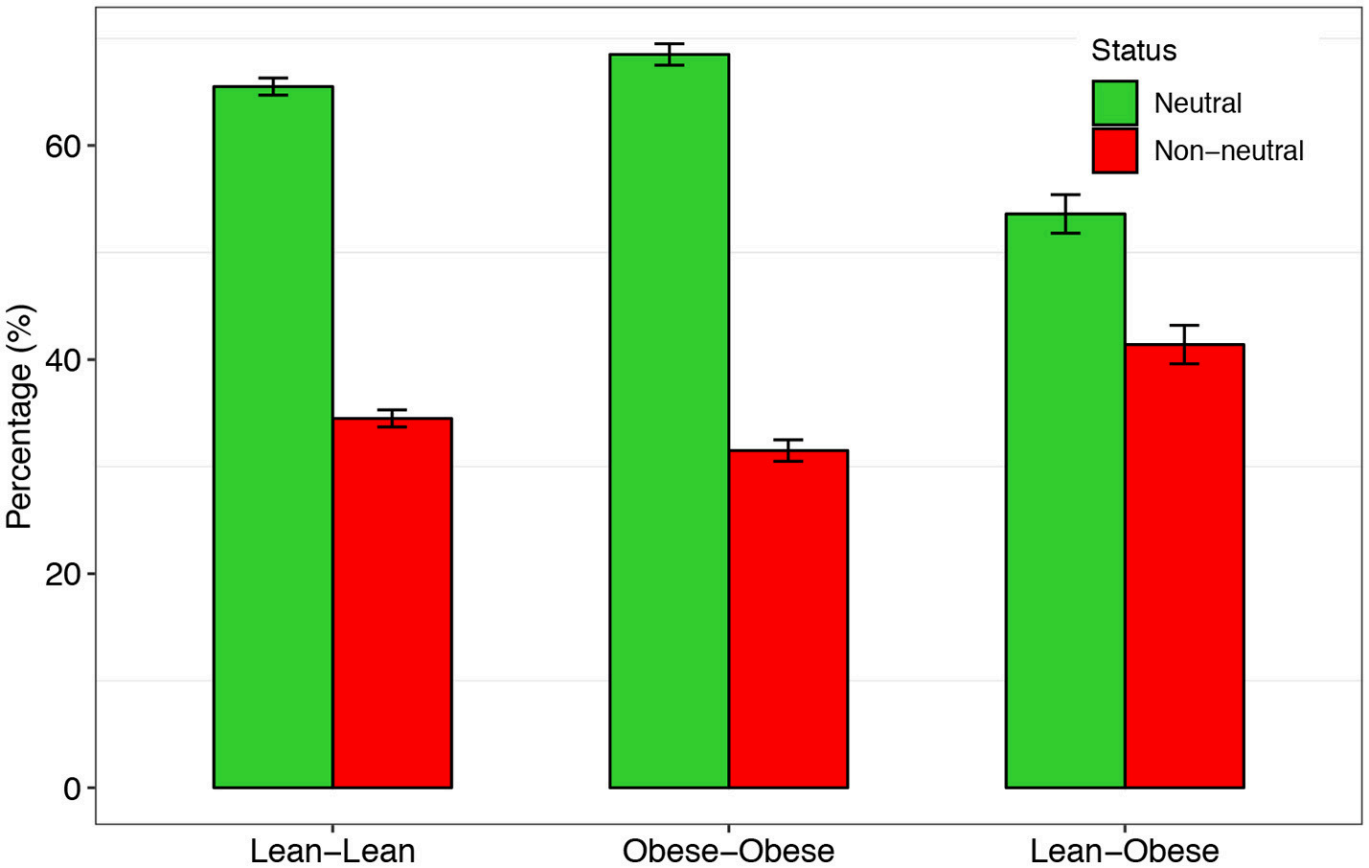
The percentages of the neutral (green) and non-neutral (red) species, respectively, in the three regimes designed for testing Sloan's neutral theory.

As shown in Table 2, there are 65.5 and 68.5% of the species that satisfied Sloan's neutral theory in the gut microbial communities of the lean and obese treatment, respectively. In other words, in more than a half of the species in the gut microbiome, stochastic neutral effects are significant. In addition, there were no significant differences in the percentage of neutral species between the obesity and lean treatments (*t*-test: *p* > 0.05, Figure 2). We also tested Sloan's neutral model by treating the lean treatment as source community and the obese treatment as the destination community, and the percentage of neutral species is slightly less (58.6%) than those of the lean or obese treatment alone.

The results from testing Sloan's neutral model seemed to be in conflict with the results from testing Hubbell's neutral model. Why are there more than a half of neutral species in a non-neutral community? The apparent contradiction can be easily resolved if we recall that Hubbell's neutral theory is tested at the whole community level, and a portion of the non-neutral species in a community is sufficient to change the behavior of the whole community. Since Sloan's model tests the neutrality of individual species, theoretically, only if all species in a community pass Sloan's neutrality test, then it should be guaranteed that the whole community is neutral in terms of Hubbell's model. In our study, there were still more than 1/3 of species that clearly demonstrated non-neutral behavior, hence, the results from both the neutral models not only do not contradict with each other, but also present complementary insights for understanding the community assembly mechanisms of the human gut microbiome.

### Conclusions and discussion

In summary, in this study, we applied both Hubbell's and Sloan's neutral theory models to test the influence of obesity on the gut microbiome assembly from both community and species perspectives. At community level, we found that all 283 but 2 gut microbial community samples we tested failed to pass the test of Hubbell's neutral theory, and obesity did not affect the test results. We conclude that the gut microbiome, as a whole, is not neutral and is governed by deterministic host effects. Obesity does not play a significant role in determining the rules (mechanisms) of gut microbiome assembly. From a species perspective, although more than a half of the species in gut microbiome were neutral according to Sloan's neutral model, it is the minority (~1/3 of species) that ultimately determined the behavior' of community as a whole. Our findings suggest that gut microbial community is a world consisting of both neutral and non-neutral species, whose collective behavior (i.e., assembly and diversity maintenance mechanisms) is determined by the non-neutral ones. Furthermore, we failed to detect a significant influence of the obesity on neutrality at either species or community scale.

Testing the neutral theory models has been challenging, at least, because of the following four factors: (*i*) the availability of quality data, (*ii*) the availability of computationally efficient algorithms, (*iii*) the neutral model itself, and (*iv*) the interpretation of the test results. First, ideally, the datasets should be sampled from a metacommunity setting consistent with the model assumption, but in practice, such datasets are not easy to obtain. Second, fitting the neutral models with a truly multi-site setting (allowing the computation of variable migration rates among different local communities) was challenging until Harris et al. (2015) recent work, who developed an efficient machine-learning based algorithm. Nevertheless, the adoption of their fitting approach has been slow, possibly due to the availability of suitable datasets. For example, the datasets used in this study and Harris et al. (2015) approach cannot be utilized to test the neutral theory because we cannot assume there are exchanges of microbes (migrations) among individual subjects in ecological time and the neutral theory is largely an ecological time-scale model. Third, obviously, the neutrality assumption is overly simplified, and more recent niche-neutral hybrid models (e.g., Tang and Zhou, 2013) can help to determine the relative significance of deterministic niche forces vs. stochastic neutral forces. Yet, among the four challenges (factors), the most challenging task is to accurately interpret the results from fitting the neutral or niche-neutral hybrid models. For example, it has been suggested that neutral theory can help to determine the significance of drift, dispersal, and speciation, the three of the four key processes for driving community dynamics (the other is selection) (Vellend, 2010; Rosindell et al., 2011, 2012). The difficulty lies in the fact that processes such as dispersal may not be stochastic and instead may be asymmetric among species. In other words, dispersal may be an adaptive behavior in many cases. Therefore, to accurately interpret the results from neutrality test, additional mechanistic studies should be conducted. That said, our study has significant room to improve given the previous discussed challenges. To fully understand the mechanisms of gut microbiome assembly as well as the influences of obesity on the mechanisms, additional biomedical studies including manipulative experiments with animal models should be performed. Nevertheless, we believe that the cross-scale approach we adopted in this study should also be helpful for addressing those challenges.

## Author contributions

SM designed the study and wrote the paper. WL, YY, and YX performed the data analysis and interpretations. YS and YM participated in the data interpretation and discussion. All authors approved the submission.

### Conflict of interest statement

The authors declare that the research was conducted in the absence of any commercial or financial relationships that could be construed as a potential conflict of interest.
