# Supplementary material for: A Cross-Scale Neutral Theory Approach to the Influence of Obesity on Community Assembly of Human Gut Microbiome
Source: Front Microbiol. 2018 Oct 29;9:2320. doi: 10.3389/fmicb.2018.02320 (PMC6215851; doi:10.3389/fmicb.2018.02320)
Supplement: Supplementary file 1 [file Table_1.doc]

**Supplementary Material**

**Table S1.** The results of neutrality test for Hubbell’s neutral model with Etienne sampling formula*

| ***Group*** | ***ID*** | ***J*** | ***S*** | ***θ*** | ***m*** | ***Log(L_0_)*** | ***Log(L_1_)*** | ***q-value*** | ***p-value*** |
| --- | --- | --- | --- | --- | --- | --- | --- | --- | --- |
| Lean | TS109.2_299100 | 1331 | 196 | 63.14 | 0.99997 | -79.15 | -68.68 | 20.94 | < 0.0001 |
|  | TS109_299099 | 1605 | 216 | 67.12 | 0.98829 | -99.97 | -76.24 | 47.46 | < 0.0001 |
|  | TS110.2_299101 | 3004 | 215 | 54.72 | 0.74451 | -127.86 | -116.53 | 22.66 | < 0.0001 |
|  | TS110_299019 | 1672 | 204 | 60.85 | 0.98801 | -95.18 | -80.38 | 29.59 | < 0.0001 |
|  | TS111.2_298964 | 2084 | 269 | 81.96 | 0.99998 | -96.57 | -87.32 | 18.50 | < 0.0001 |
|  | TS111_299174 | 2717 | 311 | 90.71 | 0.99937 | -110.61 | -100.41 | 20.41 | < 0.0001 |
|  | TS1.2_299121 | 1012 | 164 | 55.22 | 0.99443 | -67.08 | -58.68 | 16.79 | < 0.0001 |
|  | TS124.2_299092 | 1831 | 181 | 49.65 | 0.99998 | -106.31 | -87.87 | 36.90 | < 0.0001 |
|  | TS124_298945 | 1924 | 227 | 66.70 | 0.99999 | -114.20 | -86.42 | 55.55 | < 0.0001 |
|  | TS127.2_299143 | 1633 | 257 | 85.45 | 0.99993 | -82.35 | -72.92 | 18.87 | < 0.0001 |
|  | TS127_299133 | 2665 | 277 | 77.83 | 0.99934 | -119.81 | -102.80 | 34.02 | < 0.0001 |
|  | TS128.2_299216 | 2451 | 298 | 88.59 | 0.99996 | -113.14 | -94.88 | 36.52 | < 0.0001 |
|  | TS128_299117 | 3465 | 265 | 66.65 | 0.99990 | -133.92 | -124.01 | 19.83 | < 0.0001 |
|  | TS129.2_299037 | 1902 | 209 | 59.68 | 0.99998 | -111.06 | -86.88 | 48.36 | < 0.0001 |
|  | TS129_299204 | 1759 | 251 | 79.79 | 0.99995 | -103.76 | -78.17 | 51.19 | < 0.0001 |
|  | TS1_299139 | 4700 | 341 | 84.15 | 0.99983 | -174.70 | -143.01 | 63.37 | < 0.0001 |
|  | TS13.2_298954 | 1127 | 219 | 80.68 | 0.99710 | -75.13 | -57.59 | 35.08 | < 0.0001 |
|  | TS13_299018 | 6385 | 407 | 96.66 | 0.99998 | -200.47 | -169.03 | 62.88 | < 0.0001 |
|  | TS14_299212 | 12700 | 482 | 99.14 | 0.99995 | -273.02 | -249.19 | 47.64 | < 0.0001 |
|  | TS154.2_299022 | 1152 | 156 | 48.51 | 0.99975 | -70.25 | -65.95 | 8.60 | 0.0034 |
|  | TS155.2_298990 | 1802 | 204 | 58.84 | 0.99977 | -97.87 | -85.38 | 24.98 | < 0.0001 |
|  | TS155_299082 | 1729 | 180 | 50.40 | 0.99998 | -112.53 | -84.57 | 55.91 | < 0.0001 |
|  | TS16_299068 | 3886 | 267 | 64.82 | 0.99912 | -149.68 | -132.42 | 34.52 | < 0.0001 |
|  | TS163.2_299114 | 2098 | 232 | 66.69 | 0.99660 | -120.15 | -91.29 | 57.71 | < 0.0001 |
|  | TS163_299072 | 1316 | 190 | 60.77 | 0.99997 | -81.04 | -68.85 | 24.38 | < 0.0001 |
|  | TS164.2_299007 | 2518 | 223 | 58.92 | 0.99837 | -132.97 | -104.25 | 57.44 | < 0.0001 |
|  | TS164_299125 | 2107 | 184 | 48.39 | 0.99994 | -114.98 | -95.85 | 38.25 | < 0.0001 |
|  | TS165.2_299138 | 2250 | 216 | 58.62 | 0.99996 | -106.70 | -97.25 | 18.90 | < 0.0001 |
|  | TS165_298951 | 2076 | 232 | 66.67 | 0.99998 | -106.98 | -91.04 | 31.87 | < 0.0001 |
|  | TS17_299122 | 580 | 152 | 66.76 | 0.99999 | -45.56 | -38.12 | 14.88 | 0.0001 |
|  | TS180_299063 | 1896 | 199 | 55.88 | 0.99999 | -103.78 | -87.73 | 32.10 | < 0.0001 |
|  | TS184_299203 | 1648 | 161 | 44.07 | 0.99990 | -94.97 | -83.70 | 22.56 | < 0.0001 |
|  | TS185.2_298957 | 2163 | 247 | 71.71 | 0.99998 | -106.77 | -91.67 | 30.21 | < 0.0001 |
|  | TS185_299148 | 2229 | 257 | 74.78 | 0.99998 | -109.89 | -93.24 | 33.30 | < 0.0001 |
|  | TS193.2_299051 | 1467 | 227 | 74.81 | 0.99994 | -91.71 | -70.61 | 42.21 | < 0.0001 |
|  | TS193_299075 | 1206 | 181 | 58.89 | 0.99998 | -77.43 | -65.62 | 23.63 | < 0.0001 |
|  | TS194.2_299056 | 1366 | 191 | 60.13 | 0.99994 | -85.41 | -71.07 | 28.68 | < 0.0001 |
|  | TS194_299181 | 1654 | 190 | 55.24 | 0.99997 | -90.99 | -81.07 | 19.83 | < 0.0001 |
|  | TS195.2_298978 | 2142 | 217 | 60.07 | 0.99986 | -107.99 | -93.71 | 28.55 | < 0.0001 |
|  | TS195_299150 | 1884 | 217 | 63.22 | 0.99999 | -114.14 | -85.91 | 56.45 | < 0.0001 |
|  | TS2.2_299116 | 1314 | 203 | 66.87 | 1.00000 | -92.20 | -67.68 | 49.03 | < 0.0001 |
|  | TS22_299221 | 3915 | 341 | 89.66 | 0.99991 | -153.61 | -126.83 | 53.56 | < 0.0001 |
|  | TS2_299130 | 10013 | 444 | 95.31 | 0.99973 | -245.22 | -219.35 | 51.75 | < 0.0001 |
|  | TS23_299159 | 1383 | 185 | 57.14 | 0.99994 | -81.04 | -71.68 | 18.72 | < 0.0001 |
|  | TS25.2_299001 | 3205 | 289 | 76.70 | 0.99983 | -140.16 | -115.55 | 49.22 | < 0.0001 |
|  | TS25_299024 | 7101 | 407 | 93.44 | 0.99985 | -219.52 | -179.88 | 79.28 | < 0.0001 |
|  | TS26.2_299028 | 3527 | 323 | 86.21 | 0.99994 | -157.91 | -120.03 | 75.77 | < 0.0001 |
|  | TS26_299185 | 12849 | 419 | 82.71 | 0.99720 | -269.11 | -253.99 | 30.24 | < 0.0001 |
|  | TS30.2_299050 | 2256 | 203 | 53.93 | 0.99998 | -113.86 | -98.50 | 30.72 | < 0.0001 |
|  | TS31.2_299187 | 4384 | 289 | 69.18 | 0.99993 | -166.78 | -141.44 | 50.67 | < 0.0001 |
|  | TS31_299055 | 15291 | 479 | 93.68 | 0.99889 | -284.27 | -276.43 | 15.68 | 0.0001 |
|  | TS32.2_298970 | 3164 | 308 | 84.50 | 0.99936 | -129.65 | -112.23 | 34.84 | < 0.0001 |
|  | TS32_299074 | 7154 | 367 | 81.67 | 0.99988 | -198.78 | -183.56 | 30.45 | < 0.0001 |
|  | TS4.2_298988 | 2203 | 209 | 56.51 | 0.99998 | -116.27 | -96.42 | 39.71 | < 0.0001 |
|  | TS4_299175 | 6881 | 285 | 59.91 | 0.99997 | -196.11 | -182.99 | 26.24 | < 0.0001 |
|  | TS5.2_298961 | 2507 | 202 | 51.58 | 0.99997 | -119.98 | -105.51 | 28.94 | < 0.0001 |
|  | TS5_299045 | 11799 | 456 | 93.95 | 0.99728 | -270.67 | -240.35 | 60.64 | < 0.0001 |
|  | TS7.2_299010 | 2313 | 230 | 63.71 | 0.98789 | -109.57 | -97.46 | 24.22 | < 0.0001 |
|  | TS7_299061 | 9055 | 484 | 109.46 | 0.99974 | -238.07 | -204.28 | 67.57 | < 0.0001 |
|  | TS8.2_299029 | 416 | 127 | 61.95 | 0.99996 | -33.47 | -30.76 | 5.42 | 0.0199 |
|  | TS8_299198 | 13047 | 492 | 101.23 | 0.99973 | -274.63 | -252.80 | 43.65 | < 0.0001 |
| Obese | TS100.2_298944 | 1921 | 266 | 83.51 | 0.99991 | -95.26 | -82.24 | 26.05 | < 0.0001 |
|  | TS100_298999 | 2723 | 292 | 82.64 | 0.99997 | -129.96 | -102.61 | 54.70 | < 0.0001 |
|  | TS101.2_299207 | 1882 | 216 | 62.82 | 0.99996 | -99.50 | -85.68 | 27.63 | < 0.0001 |
|  | TS10.2_299108 | 793 | 130 | 43.98 | 0.99975 | -61.79 | -52.35 | 18.88 | < 0.0001 |
|  | TS10_299123 | 1923 | 197 | 54.75 | 0.99994 | -101.30 | -88.97 | 24.66 | < 0.0001 |
|  | TS103.2_299213 | 2434 | 253 | 70.90 | 0.99998 | -113.01 | -99.27 | 27.48 | < 0.0001 |
|  | TS103_299049 | 1132 | 159 | 50.14 | 0.99997 | -77.92 | -64.58 | 26.68 | < 0.0001 |
|  | TS104_299154 | 1591 | 181 | 52.35 | 0.99998 | -95.08 | -80.13 | 29.89 | < 0.0001 |
|  | TS105.2_299038 | 1776 | 137 | 34.47 | 0.99998 | -92.84 | -89.65 | 6.38 | 0.0115 |
|  | TS105_299210 | 1513 | 165 | 47.02 | 0.98657 | -83.06 | -78.40 | 9.31 | 0.0023 |
|  | TS106.2_299179 | 1819 | 196 | 55.63 | 0.99995 | -103.78 | -86.09 | 35.37 | < 0.0001 |
|  | TS106_299035 | 2072 | 220 | 61.99 | 0.99994 | -101.50 | -91.90 | 19.20 | < 0.0001 |
|  | TS107.2_298960 | 3449 | 282 | 73.64 | 0.92234 | -140.67 | -120.68 | 39.99 | < 0.0001 |
|  | TS107_299042 | 2347 | 222 | 60.04 | 0.99998 | -109.52 | -99.52 | 19.99 | < 0.0001 |
|  | TS11.2_299208 | 2119 | 176 | 45.51 | 0.98708 | -102.01 | -96.45 | 11.11 | 0.0009 |
|  | TS11_298996 | 2118 | 193 | 51.55 | 0.99992 | -99.61 | -95.75 | 7.71 | 0.0055 |
|  | TS115.2_299043 | 1494 | 248 | 84.50 | 0.99994 | -80.57 | -68.92 | 23.29 | < 0.0001 |
|  | TS115_298998 | 1973 | 286 | 91.69 | 0.99990 | -92.83 | -81.06 | 23.54 | < 0.0001 |
|  | TS116.2_299078 | 1784 | 272 | 89.29 | 0.99998 | -99.57 | -76.06 | 47.02 | < 0.0001 |
|  | TS116_299165 | 3666 | 318 | 83.52 | 0.99998 | -159.11 | -123.57 | 71.07 | < 0.0001 |
|  | TS117.2_299030 | 2367 | 226 | 61.33 | 0.99998 | -111.87 | -99.71 | 24.32 | < 0.0001 |
|  | TS117_298949 | 2610 | 241 | 64.53 | 0.99903 | -117.74 | -104.70 | 26.07 | < 0.0001 |
|  | TS118.2_298966 | 2231 | 246 | 70.46 | 0.99998 | -111.88 | -93.25 | 37.26 | < 0.0001 |
|  | TS118_299163 | 1823 | 215 | 63.09 | 0.99994 | -104.27 | -83.92 | 40.70 | < 0.0001 |
|  | TS119.2_299032 | 2976 | 273 | 73.29 | 0.99934 | -140.33 | -110.82 | 59.01 | < 0.0001 |
|  | TS119_299110 | 2298 | 234 | 65.10 | 0.99980 | -123.25 | -96.90 | 52.69 | < 0.0001 |
|  | TS120.2_299136 | 2250 | 276 | 82.48 | 0.99998 | -109.34 | -91.33 | 36.03 | < 0.0001 |
|  | TS12.2_299176 | 1477 | 163 | 46.56 | 0.99994 | -94.43 | -77.23 | 34.40 | < 0.0001 |
|  | TS12_299104 | 1837 | 171 | 45.96 | 0.99995 | -108.41 | -88.92 | 38.99 | < 0.0001 |
|  | TS126.2_298986 | 2174 | 242 | 69.48 | 0.99999 | -128.95 | -92.93 | 72.04 | < 0.0001 |
|  | TS126_299046 | 1382 | 258 | 93.20 | 0.99961 | -103.29 | -63.77 | 79.04 | < 0.0001 |
|  | TS130.2_298955 | 2864 | 196 | 47.54 | 0.99958 | -125.53 | -114.83 | 21.40 | < 0.0001 |
|  | TS130_299178 | 2683 | 174 | 41.51 | 0.99890 | -124.83 | -111.66 | 26.35 | < 0.0001 |
|  | TS131.2_298993 | 2809 | 188 | 45.28 | 0.99994 | -127.48 | -113.73 | 27.51 | < 0.0001 |
|  | TS131_299040 | 2704 | 216 | 55.17 | 0.99308 | -117.71 | -109.82 | 15.78 | 0.0001 |
|  | TS132.2_299033 | 3271 | 276 | 71.67 | 0.99997 | -139.33 | -118.29 | 42.07 | < 0.0001 |
|  | TS132_299044 | 2111 | 207 | 56.74 | 0.99997 | -110.03 | -94.64 | 30.77 | < 0.0001 |
|  | TS133.2_299076 | 3309 | 240 | 59.23 | 0.99990 | -141.33 | -121.98 | 38.70 | < 0.0001 |
|  | TS133_299149 | 1898 | 218 | 63.51 | 0.98812 | -106.73 | -86.81 | 39.84 | < 0.0001 |
|  | TS134.2_298989 | 2114 | 209 | 57.44 | 0.99998 | -106.88 | -93.61 | 26.53 | < 0.0001 |
|  | TS134_299196 | 1124 | 191 | 65.85 | 0.99998 | -69.52 | -60.94 | 17.15 | < 0.0001 |
|  | TS135.2_299222 | 2067 | 203 | 55.65 | 0.99999 | -104.59 | -93.16 | 22.85 | < 0.0001 |
|  | TS135_299142 | 2517 | 253 | 69.87 | 0.99994 | -120.34 | -101.11 | 38.46 | < 0.0001 |
|  | TS136.2_298983 | 3107 | 366 | 107.72 | 0.99993 | -123.87 | -105.18 | 37.38 | < 0.0001 |
|  | TS137.2_299107 | 2188 | 243 | 69.69 | 0.99995 | -105.76 | -92.46 | 26.59 | < 0.0001 |
|  | TS137_298959 | 3017 | 247 | 63.53 | 0.99998 | -125.04 | -114.36 | 21.35 | < 0.0001 |
|  | TS138.2_299162 | 1092 | 170 | 56.26 | 0.99994 | -80.85 | -61.53 | 38.64 | < 0.0001 |
|  | TS138_299141 | 2653 | 261 | 71.56 | 0.99996 | -127.40 | -103.60 | 47.60 | < 0.0001 |
|  | TS139.2_299031 | 2368 | 263 | 75.55 | 0.99906 | -117.07 | -96.20 | 41.74 | < 0.0001 |
|  | TS139_299195 | 2340 | 260 | 74.75 | 0.99858 | -103.08 | -95.37 | 15.43 | 0.0001 |
|  | TS140.2_299087 | 1989 | 209 | 58.76 | 0.99999 | -114.59 | -90.01 | 49.15 | < 0.0001 |
|  | TS140_298995 | 1903 | 261 | 81.69 | 0.99997 | -100.49 | -81.76 | 37.46 | < 0.0001 |
|  | TS141.2_299017 | 2399 | 279 | 81.58 | 0.99999 | -116.64 | -94.95 | 43.38 | < 0.0001 |
|  | TS141_299084 | 1632 | 228 | 72.03 | 0.98847 | -97.46 | -76.23 | 42.46 | < 0.0001 |
|  | TS142.2_298963 | 2779 | 231 | 59.73 | 0.99998 | -126.32 | -110.02 | 32.59 | < 0.0001 |
|  | TS142_299170 | 1820 | 234 | 71.31 | 0.99997 | -95.89 | -82.28 | 27.22 | < 0.0001 |
|  | TS143.2_299034 | 2918 | 264 | 70.37 | 0.99975 | -133.11 | -111.17 | 43.88 | < 0.0001 |
|  | TS143_299098 | 3051 | 312 | 86.91 | 0.99909 | -129.77 | -109.35 | 40.84 | < 0.0001 |
|  | TS144.2_299166 | 3394 | 304 | 80.73 | 0.99998 | -147.24 | -118.34 | 57.80 | < 0.0001 |
|  | TS144_298946 | 2149 | 234 | 66.58 | 0.99998 | -109.36 | -92.75 | 33.22 | < 0.0001 |
|  | TS145.2_299151 | 2655 | 182 | 44.07 | 0.99996 | -116.52 | -110.54 | 11.96 | 0.0005 |
|  | TS145_298950 | 1991 | 167 | 43.31 | 0.99992 | -97.61 | -93.09 | 9.04 | 0.0026 |
|  | TS146.2_299211 | 2662 | 225 | 58.53 | 0.99994 | -127.45 | -107.59 | 39.73 | < 0.0001 |
|  | TS146_299014 | 2570 | 220 | 57.35 | 0.99998 | -121.90 | -106.12 | 31.58 | < 0.0001 |
|  | TS147_299111 | 2731 | 265 | 72.58 | 0.99933 | -120.19 | -105.93 | 28.52 | < 0.0001 |
|  | TS148_299000 | 4561 | 321 | 78.44 | 0.99983 | -158.59 | -142.27 | 32.65 | < 0.0001 |
|  | TS149_298994 | 6980 | 392 | 89.75 | 0.99997 | -202.40 | -180.14 | 44.53 | < 0.0001 |
|  | TS150_299173 | 6126 | 354 | 81.55 | 0.99997 | -192.49 | -169.11 | 46.77 | < 0.0001 |
|  | TS151.2_298991 | 2531 | 220 | 57.68 | 0.99996 | -129.76 | -104.81 | 49.91 | < 0.0001 |
|  | TS151_299215 | 3022 | 272 | 72.21 | 0.99996 | -131.23 | -111.78 | 38.91 | < 0.0001 |
|  | TS152.2_299097 | 2073 | 226 | 64.45 | 0.99998 | -112.57 | -90.93 | 43.29 | < 0.0001 |
|  | TS152_299135 | 2575 | 213 | 54.71 | 0.99967 | -123.79 | -106.60 | 34.39 | < 0.0001 |
|  | TS156.2_299036 | 1544 | 240 | 79.38 | 0.99968 | -85.41 | -71.52 | 27.78 | < 0.0001 |
|  | TS156_299155 | 1331 | 202 | 65.97 | 0.99995 | -85.08 | -68.19 | 33.77 | < 0.0001 |
|  | TS160.2_299131 | 1581 | 200 | 60.47 | 0.99998 | -109.75 | -77.15 | 65.21 | < 0.0001 |
|  | TS160_298976 | 1704 | 200 | 58.69 | 0.99998 | -99.67 | -81.71 | 35.91 | < 0.0001 |
|  | TS161_299188 | 1719 | 160 | 42.89 | 0.99998 | -90.75 | -86.36 | 8.79 | 0.0030 |
|  | TS162.2_299140 | 3346 | 149 | 31.87 | 0.99995 | -116.99 | -125.13 | 16.29 | 0.0001 |
|  | TS162_299124 | 3144 | 144 | 30.99 | 0.99995 | -114.37 | -120.89 | 13.03 | 0.0003 |
|  | TS166.2_298980 | 2427 | 231 | 62.47 | 0.99996 | -114.82 | -100.72 | 28.21 | < 0.0001 |
|  | TS166_299085 | 2793 | 241 | 63.09 | 0.99915 | -131.75 | -108.59 | 46.32 | < 0.0001 |
|  | TS167.2_299004 | 3195 | 267 | 69.04 | 0.99935 | -137.35 | -118.08 | 38.54 | < 0.0001 |
|  | TS167_299073 | 2129 | 209 | 57.18 | 0.99996 | -102.99 | -93.60 | 18.78 | < 0.0001 |
|  | TS168.2_298985 | 3137 | 300 | 81.39 | 0.99779 | -133.10 | -113.97 | 38.25 | < 0.0001 |
|  | TS168_299115 | 2057 | 254 | 76.01 | 0.99972 | -109.69 | -87.34 | 44.70 | < 0.0001 |
|  | TS169.2_298975 | 2658 | 332 | 99.71 | 0.99959 | -104.13 | -97.36 | 13.55 | 0.0002 |
|  | TS169_298977 | 3086 | 351 | 101.63 | 0.99996 | -124.03 | -106.62 | 34.82 | < 0.0001 |
|  | TS170.2_299205 | 2131 | 214 | 59.13 | 0.99997 | -114.45 | -93.78 | 41.34 | < 0.0001 |
|  | TS178.2_299168 | 2027 | 246 | 73.16 | 0.99997 | -100.44 | -87.48 | 25.92 | < 0.0001 |
|  | TS178_299126 | 1854 | 216 | 63.17 | 0.99999 | -114.76 | -86.08 | 57.35 | < 0.0001 |
|  | TS179.2_299016 | 2319 | 262 | 75.76 | 0.99998 | -120.09 | -95.23 | 49.72 | < 0.0001 |
|  | TS179_298987 | 1696 | 200 | 58.70 | 0.99993 | -84.71 | -81.63 | 6.15 | 0.0132 |
|  | TS181.2_299083 | 1898 | 194 | 53.98 | 0.99998 | -98.07 | -88.75 | 18.64 | < 0.0001 |
|  | TS181_299047 | 2374 | 250 | 70.24 | 0.99996 | -106.73 | -97.62 | 18.21 | < 0.0001 |
|  | TS182.2_299113 | 4508 | 361 | 92.15 | 0.99919 | -166.02 | -138.27 | 55.49 | < 0.0001 |
|  | TS183.2_299060 | 1456 | 172 | 50.47 | 0.99999 | -97.76 | -75.81 | 43.90 | < 0.0001 |
|  | TS183_298979 | 1642 | 181 | 51.76 | 0.99999 | -95.40 | -81.92 | 26.96 | < 0.0001 |
|  | TS190_299218 | 2403 | 273 | 78.99 | 0.99908 | -108.57 | -95.84 | 25.45 | < 0.0001 |
|  | TS191_299146 | 2196 | 194 | 51.10 | 0.99997 | -111.11 | -97.60 | 27.02 | < 0.0001 |
|  | TS19.2_298956 | 3008 | 202 | 48.69 | 0.99997 | -124.65 | -117.49 | 14.33 | 0.0002 |
|  | TS19_298958 | 7578 | 368 | 80.79 | 0.99993 | -220.16 | -189.79 | 60.74 | < 0.0001 |
|  | TS20.2_299090 | 3203 | 247 | 62.22 | 0.99997 | -138.35 | -119.15 | 38.39 | < 0.0001 |
|  | TS20_299059 | 13649 | 423 | 82.47 | 0.99720 | -276.81 | -262.50 | 28.63 | < 0.0001 |
|  | TS21.2_299079 | 3837 | 273 | 67.00 | 0.99994 | -141.47 | -131.59 | 19.74 | < 0.0001 |
|  | TS21_299189 | 11689 | 469 | 98.05 | 0.99973 | -253.03 | -238.44 | 29.19 | < 0.0001 |
|  | TS27.2_299025 | 1450 | 223 | 73.37 | 0.99999 | -76.68 | -69.99 | 13.38 | 0.0003 |
|  | TS27_299223 | 8885 | 393 | 83.98 | 0.99997 | -233.71 | -207.93 | 51.55 | < 0.0001 |
|  | TS28_299120 | 5212 | 359 | 87.26 | 0.99998 | -180.81 | -152.04 | 57.53 | < 0.0001 |
|  | TS29_298974 | 1834 | 209 | 60.50 | 0.99994 | -98.68 | -84.45 | 28.47 | < 0.0001 |
|  | TS34_299217 | 1165 | 183 | 60.72 | 0.99996 | -76.02 | -63.29 | 25.46 | < 0.0001 |
|  | TS35_298981 | 2621 | 283 | 80.30 | 0.99908 | -122.06 | -101.30 | 41.52 | < 0.0001 |
|  | TS37.2_299096 | 3530 | 292 | 75.20 | 0.99982 | -145.40 | -122.77 | 45.27 | < 0.0001 |
|  | TS37_299147 | 2351 | 231 | 63.36 | 0.99998 | -121.13 | -97.99 | 46.28 | < 0.0001 |
|  | TS39.2_299009 | 1563 | 230 | 74.13 | 0.99994 | -80.19 | -73.87 | 12.64 | 0.0004 |
|  | TS39_299167 | 4203 | 317 | 79.46 | 0.99997 | -156.57 | -134.51 | 44.11 | < 0.0001 |
|  | TS43_299095 | 1992 | 242 | 71.89 | 0.99998 | -102.86 | -87.19 | 31.34 | < 0.0001 |
|  | TS44_299183 | 774 | 122 | 40.51 | 0.99998 | -54.88 | -52.47 | 4.81 | 0.0282 |
|  | TS49.2_299169 | 2886 | 221 | 55.61 | 0.99995 | -132.23 | -113.09 | 38.27 | < 0.0001 |
|  | TS49_298982 | 8845 | 371 | 78.07 | 0.99985 | -234.48 | -207.62 | 53.73 | < 0.0001 |
|  | TS50.2_299158 | 2832 | 211 | 52.52 | 0.99992 | -126.23 | -113.02 | 26.42 | < 0.0001 |
|  | TS50_299027 | 9094 | 296 | 58.48 | 0.97931 | -227.38 | -213.07 | 28.62 | < 0.0001 |
|  | TS51.2_299062 | 2296 | 195 | 50.78 | 0.99990 | -109.43 | -100.23 | 18.42 | < 0.0001 |
|  | TS51_299145 | 9775 | 423 | 90.20 | 0.99973 | -237.19 | -216.52 | 41.33 | < 0.0001 |
|  | TS55.2_298952 | 4127 | 251 | 58.78 | 0.99996 | -149.10 | -137.92 | 22.37 | < 0.0001 |
|  | TS55_298947 | 3117 | 253 | 64.85 | 0.99998 | -137.90 | -116.27 | 43.26 | < 0.0001 |
|  | TS56.2_299192 | 3041 | 153 | 33.88 | 0.99995 | -123.28 | -119.61 | 7.34 | 0.0068 |
|  | TS56_299021 | 4196 | 278 | 66.80 | 0.99997 | -156.21 | -138.28 | 35.86 | < 0.0001 |
|  | TS57.2_299112 | 1648 | 134 | 34.23 | 0.99948 | -100.24 | -86.13 | 28.22 | < 0.0001 |
|  | TS57_299197 | 6670 | 153 | 27.75 | 0.99988 | -155.69 | -172.76 | 34.15 | < 0.0001 |
|  | TS61.2_299186 | 2845 | 269 | 72.74 | 0.99918 | -131.79 | -108.16 | 47.25 | < 0.0001 |
|  | TS61_299129 | 494 | 110 | 43.63 | 0.99998 | -47.88 | -38.82 | 18.11 | < 0.0001 |
|  | TS62.2_299088 | 2794 | 266 | 72.05 | 0.99997 | -130.01 | -107.31 | 45.42 | < 0.0001 |
|  | TS62_299066 | 1612 | 166 | 46.26 | 0.99982 | -92.91 | -82.18 | 21.47 | < 0.0001 |
|  | TS6.2_299094 | 3198 | 229 | 56.34 | 0.99913 | -142.40 | -120.51 | 43.78 | < 0.0001 |
|  | TS6_299086 | 6261 | 364 | 84.22 | 0.99994 | -215.48 | -169.54 | 91.88 | < 0.0001 |
|  | TS63.2_299109 | 1460 | 209 | 66.52 | 0.99994 | -81.70 | -72.16 | 19.09 | < 0.0001 |
|  | TS63_299005 | 1492 | 175 | 51.27 | 0.99998 | -93.29 | -76.81 | 32.96 | < 0.0001 |
|  | TS65.2_299065 | 2361 | 276 | 80.94 | 0.99996 | -115.61 | -94.49 | 42.23 | < 0.0001 |
|  | TS65_299182 | 1677 | 229 | 71.52 | 0.99993 | -84.79 | -77.54 | 14.51 | 0.0001 |
|  | TS66.2_299157 | 7698 | 465 | 108.82 | 0.99997 | -215.62 | -185.51 | 60.23 | < 0.0001 |
|  | TS66_299194 | 2364 | 216 | 57.68 | 0.99998 | -112.15 | -100.64 | 23.01 | < 0.0001 |
|  | TS67.2_299127 | 1670 | 230 | 71.98 | 0.99994 | -93.33 | -77.60 | 31.46 | < 0.0001 |
|  | TS67_299039 | 715 | 156 | 61.22 | 0.99998 | -53.98 | -46.07 | 15.81 | 0.0001 |
|  | TS68.2_298971 | 1825 | 276 | 90.24 | 0.99998 | -98.21 | -77.83 | 40.76 | < 0.0001 |
|  | TS68_299202 | 2774 | 251 | 66.81 | 0.99996 | -121.31 | -108.46 | 25.69 | < 0.0001 |
|  | TS69.2_298972 | 2031 | 254 | 76.48 | 0.99997 | -102.20 | -87.32 | 29.76 | < 0.0001 |
|  | TS69_299058 | 3424 | 219 | 52.07 | 0.99997 | -141.98 | -125.88 | 32.20 | < 0.0001 |
|  | TS70.2_299052 | 1751 | 239 | 74.75 | 0.98856 | -93.61 | -79.03 | 29.17 | < 0.0001 |
|  | TS70_299093 | 1344 | 207 | 68.15 | 0.99999 | -82.90 | -67.60 | 30.60 | < 0.0001 |
|  | TS71.2_299080 | 1880 | 277 | 89.51 | 0.99999 | -105.42 | -78.91 | 53.02 | < 0.0001 |
|  | TS71_298968 | 2230 | 282 | 85.32 | 0.99997 | -108.83 | -89.28 | 39.10 | < 0.0001 |
|  | TS72.2_299026 | 279 | 97 | 52.36 | 0.99998 | -27.77 | -24.67 | 6.20 | 0.0128 |
|  | TS72_299209 | 1237 | 205 | 69.75 | 0.99995 | -71.60 | -64.55 | 14.10 | 0.0002 |
|  | TS73_299077 | 2572 | 199 | 50.09 | 0.99996 | -119.12 | -107.96 | 22.32 | < 0.0001 |
|  | TS74.2_299132 | 1338 | 255 | 93.17 | 0.99971 | -73.34 | -61.41 | 23.87 | < 0.0001 |
|  | TS74_299177 | 1855 | 278 | 90.48 | 0.99999 | -96.97 | -77.97 | 37.99 | < 0.0001 |
|  | TS75.2_298948 | 1676 | 148 | 38.95 | 0.99997 | -86.33 | -85.81 | 1.05 | 0.3052 |
|  | TS75_299153 | 1259 | 118 | 31.74 | 0.99998 | -76.40 | -74.05 | 4.71 | 0.0300 |
|  | TS76.2_299161 | 3102 | 321 | 90.08 | 0.99937 | -129.83 | -109.22 | 41.23 | < 0.0001 |
|  | TS76_299071 | 2489 | 267 | 75.55 | 0.99996 | -122.56 | -99.27 | 46.58 | < 0.0001 |
|  | TS78.2_299070 | 2045 | 191 | 51.34 | 0.99998 | -105.52 | -93.77 | 23.50 | < 0.0001 |
|  | TS78_299102 | 1108 | 157 | 49.67 | 0.99996 | -79.22 | -64.07 | 30.31 | < 0.0001 |
|  | TS82.2_299057 | 1316 | 202 | 66.28 | 0.99935 | -79.68 | -67.89 | 23.57 | < 0.0001 |
|  | TS82_299048 | 1248 | 177 | 56.08 | 0.99999 | -89.38 | -67.58 | 43.62 | < 0.0001 |
|  | TS83.2_299171 | 2651 | 243 | 65.05 | 0.99853 | -118.14 | -105.26 | 25.76 | < 0.0001 |
|  | TS83_299134 | 1354 | 189 | 59.46 | 0.99994 | -87.94 | -70.84 | 34.21 | < 0.0001 |
|  | TS84.2_298962 | 1407 | 223 | 74.31 | 0.99997 | -84.61 | -68.45 | 32.32 | < 0.0001 |
|  | TS84_299191 | 1535 | 170 | 48.77 | 0.99992 | -89.82 | -79.08 | 21.48 | < 0.0001 |
|  | TS86_299064 | 3387 | 303 | 80.31 | 0.99997 | -139.77 | -118.58 | 42.37 | < 0.0001 |
|  | TS87.2_298965 | 1218 | 245 | 92.01 | 0.99997 | -83.07 | -58.61 | 48.92 | < 0.0001 |
|  | TS87_299172 | 1993 | 304 | 99.68 | 0.99998 | -93.92 | -80.19 | 27.47 | < 0.0001 |
|  | TS88.2_299002 | 1763 | 210 | 61.89 | 0.99998 | -106.97 | -82.88 | 48.17 | < 0.0001 |
|  | TS88_299013 | 2118 | 183 | 47.95 | 0.99894 | -111.85 | -95.76 | 32.17 | < 0.0001 |
|  | TS89.2_299023 | 2972 | 298 | 82.25 | 0.99908 | -130.46 | -108.96 | 43.00 | < 0.0001 |
|  | TS89_299011 | 1731 | 260 | 84.67 | 0.99955 | -93.79 | -76.43 | 34.73 | < 0.0001 |
|  | TS90.2_299200 | 2216 | 278 | 83.83 | 0.99997 | -116.06 | -90.18 | 51.76 | < 0.0001 |
|  | TS90_298953 | 2189 | 278 | 84.12 | 0.99995 | -119.70 | -89.24 | 60.91 | < 0.0001 |
|  | TS91.2_299008 | 1965 | 208 | 58.55 | 0.99993 | -93.56 | -89.51 | 8.12 | 0.0044 |
|  | TS91_299152 | 547 | 143 | 62.62 | 0.99994 | -41.33 | -37.49 | 7.69 | 0.0055 |
|  | TS92.2_299118 | 2061 | 218 | 61.50 | 0.99973 | -108.73 | -91.25 | 34.97 | < 0.0001 |
|  | TS9.2_298969 | 2115 | 221 | 61.89 | 0.99996 | -101.88 | -93.16 | 17.43 | < 0.0001 |
|  | TS92_299206 | 1733 | 200 | 58.28 | 0.99996 | -88.81 | -82.92 | 11.78 | 0.0006 |
|  | TS94.2_299160 | 2028 | 241 | 71.10 | 0.99990 | -104.12 | -87.69 | 32.87 | < 0.0001 |
|  | TS94_299193 | 1553 | 195 | 58.69 | 0.99998 | -94.57 | -77.15 | 34.83 | < 0.0001 |
|  | TS95.2_299006 | 2168 | 197 | 52.42 | 0.99998 | -109.06 | -96.26 | 25.60 | < 0.0001 |
|  | TS95_299180 | 2475 | 213 | 55.45 | 0.99967 | -120.50 | -103.13 | 34.74 | < 0.0001 |
|  | TS96.2_299214 | 2461 | 278 | 80.27 | 0.99998 | -115.51 | -96.89 | 37.24 | < 0.0001 |
|  | TS96_299089 | 1257 | 192 | 63.01 | 0.99895 | -78.16 | -66.01 | 24.31 | < 0.0001 |
|  | TS97.2_299190 | 2343 | 231 | 63.78 | 0.98789 | -115.24 | -98.68 | 33.13 | < 0.0001 |
|  | TS97_299015 | 2012 | 195 | 53.14 | 0.99995 | -105.78 | -91.98 | 27.58 | < 0.0001 |
|  | TS98.2_299041 | 2431 | 181 | 45.07 | 0.99997 | -113.44 | -104.47 | 17.95 | < 0.0001 |
|  | TS98_299220 | 2602 | 177 | 42.75 | 0.99991 | -110.24 | -108.55 | 3.38 | 0.0660 |
|  | X_29TS86.2_299219 | 1409 | 206 | 66.24 | 0.99999 | -90.43 | -70.90 | 39.07 | < 0.0001 |
| Overweight | TS125.2_299067 | 1601 | 201 | 60.50 | 0.99998 | -100.43 | -77.73 | 45.40 | < 0.0001 |
|  | TS125_298984 | 2042 | 215 | 60.50 | 0.99998 | -110.20 | -90.37 | 39.66 | < 0.0001 |
|  | TS136_299091 | 2219 | 316 | 100.75 | 0.99977 | -94.92 | -85.96 | 17.94 | < 0.0001 |
|  | TS147.2_299003 | 3836 | 327 | 85.13 | 0.99983 | -150.52 | -126.66 | 47.71 | < 0.0001 |
|  | TS15.2_299128 | 2491 | 253 | 70.24 | 0.99990 | -124.46 | -100.80 | 47.32 | < 0.0001 |
|  | TS15_299020 | 12443 | 498 | 103.64 | 0.99989 | -272.50 | -246.56 | 51.87 | < 0.0001 |
|  | TS153_299184 | 3202 | 269 | 69.75 | 0.99997 | -133.43 | -117.16 | 32.54 | < 0.0001 |
|  | TS182_299201 | 5767 | 320 | 72.93 | 0.99994 | -179.58 | -164.09 | 30.99 | < 0.0001 |
|  | TS186_299199 | 1494 | 168 | 48.44 | 0.99995 | -93.40 | -78.29 | 30.22 | < 0.0001 |
|  | TS192_299012 | 3627 | 223 | 52.36 | 0.99997 | -140.66 | -129.80 | 21.73 | < 0.0001 |
|  | TS30_298973 | 5756 | 316 | 71.74 | 0.99991 | -180.34 | -164.86 | 30.96 | < 0.0001 |
|  | TS3.2_299103 | 1348 | 192 | 61.05 | 0.99996 | -84.80 | -69.79 | 30.02 | < 0.0001 |
|  | TS3_298967 | 5535 | 350 | 82.83 | 0.99997 | -188.67 | -158.25 | 60.84 | < 0.0001 |
|  | TS33.2_299054 | 1321 | 174 | 53.47 | 0.99983 | -87.84 | -69.86 | 35.97 | < 0.0001 |
|  | TS33_299224 | 11929 | 550 | 118.89 | 0.99944 | -260.24 | -236.72 | 47.03 | < 0.0001 |
|  | TS38.2_299081 | 1931 | 268 | 84.22 | 0.99998 | -98.87 | -82.08 | 33.58 | < 0.0001 |
|  | TS38_298997 | 2165 | 272 | 82.06 | 0.99997 | -108.97 | -88.15 | 41.64 | < 0.0001 |
|  | TS64.2_299164 | 2779 | 271 | 74.36 | 0.99934 | -124.27 | -106.40 | 35.75 | < 0.0001 |
|  | TS64_299137 | 2863 | 263 | 70.63 | 0.99933 | -118.95 | -109.09 | 19.73 | < 0.0001 |
|  | TS77_299105 | 1764 | 228 | 69.46 | 0.99994 | -101.73 | -80.71 | 42.03 | < 0.0001 |
|  | TS85.2_299119 | 1839 | 190 | 53.07 | 0.99998 | -100.66 | -86.13 | 29.07 | < 0.0001 |
|  | TS85_299053 | 2041 | 213 | 59.67 | 0.99995 | -102.84 | -90.20 | 25.27 | < 0.0001 |
|  | TS9_299144 | 8605 | 389 | 83.52 | 0.99721 | -226.72 | -204.49 | 44.46 | < 0.0001 |
|  | TS99.2_299069 | 2730 | 135 | 29.67 | 0.99618 | -106.20 | -112.51 | 12.61 | 0.0004 |

*It is noted that *3* communities (ID: TS104.2_299106, TS120_298992 and TS170_299156) in the obese group were omitted during computing with Etienne sampling formula, since the number of species in these communities were too few to test the neutral model.

**Table 2.** The gut microbial species passing the test of Sloan’s neutral theory in the gut microbiome of lean and obese treatments*

| **Source** | **Destination** | ***N*** | ***SD_N_*** | ***m*** | ***SD_m_*** | ***R*^2^** | ***SD_R_^2^*** | **Total** | ***SD_total_*** | **Neutral (%)** | ***SD_neutral_*** | **Non-neutral (%)** | ***SD_non-neutral_*** |
| --- | --- | --- | --- | --- | --- | --- | --- | --- | --- | --- | --- | --- | --- |
| Lean | Lean | 3629 | 203.769 | 0.043 | 0.003 | 0.416 | 0.018 | 1640 | 16.766 | 65.5 | 0.8 | 34.5 | 0.8 |
| Obese | Obese | 2543 | 226.598 | 0.063 | 0.009 | 0.472 | 0.024 | 1476 | 36.284 | 68.5 | 1.0 | 31.5 | 1.0 |
| Lean | Obese | 2586 | 236.736 | 0.032 | 0.006 | 0.296 | 0.052 | 1220 | 30.489 | 58.6 | 1.8 | 41.4 | 1.8 |

**N* is the average individuals in destination community, *SD_N_* is the standard deviation of individuals in destination community; *ms* is the immigration probability, *SD_m_* is the standard deviation of the immigration probability; *R^2^* is the goodness-of-fitting, *SD_R_^2^* is the standard deviation of the goodness-of-fitting; *total* is the total number of species in the treatment, *SD_total_* is the standard deviation of the total number of species in the treatment; *neutral* is the percentage of the species within the 95% confidence interval predicted by the neutral model, and *SD_neutral_* is the standard deviation of the percentage of neutral species; *non-neutral* is the percentage of the species deviating from the neutral model, and *SD_non-neutral_* is the standard deviation of the percentage of non-neutral species.
